# Resident intruder paradigm-induced PMDD rat model of premenstrual irritability: behavioral phenotypes, drug intervention, and biomarkers

**DOI:** 10.18632/aging.204402

**Published:** 2022-11-27

**Authors:** Mingzhou Gao, Hao Zhang, Zhan Gao, Ya Sun, Guanghao Xu, Fengqin Wei, Jieqiong Wang, Dongmei Gao

**Affiliations:** 1 Innovation Research Institute of Traditional Chinese Medicine, Shandong University of Traditional Chinese Medicine, Jinan, Shandong Province, China; 2 Experimental Center, Shandong University of Traditional Chinese Medicine, Jinan, Shandong Province, China; 3 College of Traditional Chinese Medicine, Shandong University of Traditional Chinese Medicine, Jinan, Shandong Province, China; 4 Marxism College, Shandong University of Traditional Chinese Medicine, Jinan, Shandong Province, China; 5 School of Pharmacy, Shandong University of Traditional Chinese Medicine, Jinan, Shandong Province, China

**Keywords:** PMDD, model, premenstrual irritability, ALLO, drug, biomarkers

## Abstract

Background: Premenstrual dysphoric disorder (PMDD) is high in women of childbearing age with obvious premenstrual irritability. However, reliable animal models are still lacking.

Materials and Methods: PMDD rat model of premenstrual irritability was induced by the resident-intruder paradigm (RIP). Behavioral characteristics were determined by the aggressive behavior test, elevated plus maze, open-field test, and breast width measurement. The estrous cycle in rats was artificially manipulated by bilateral ovariectomy and exogenous hormone injection to verify the model phenotype’s dependence on the estrous cycle.

Fluoxetine and Baixiangdan capsules were administered by gavage to determine the symptom improvement effect of PMDD irritability. Biomarkers in serum and brain were detected using ELISA, and GABRA4 was detected in the brain by RT-PCR and Western blot.

Results: Rat models demonstrated similar clinical characteristics as PMDD, such as premenstrual irritability and anxiety, and the above symptoms were estrous cycle-dependent. In addition, the levels of progesterone (P) and ALLO hormones decreased in the serum, hippocampus, amygdala, and frontal lobe in the NR phase. The contents of 5-HT in the brain were significantly increased, while NE and GABA contents were considerably reduced. Moreover, mRNA and protein expression of GABRA4 levels in model rats’ amygdala, hippocampus, and frontal lobe were significantly increased, while drug intervention downregulated its expression in these tissues.

Conclusion: Premenstrual irritability rat model of PMDD demonstrates a behavioral phenotype consistent with the clinical symptoms of PMDD and micro index. The increased levels of 5-HT, NE, and expression of GABRA4, as well as the decrease of GABA, P, and ALLO levels, may be critical biomarkers of the abnormal changes that occur during the pathogenesis of PMDD.

## INTRODUCTION

Premenstrual dysphoric disorder (PMDD) is a severe form of premenstrual syndrome (PMS), with clinical manifestation affecting large numbers of the female population. The symptoms of this condition are severe enough to substantially affect daily activities [1, 2]. It is characterized by significant emotional, physical, and behavioral distress during the late luteal phase that remits after menses onset [1, 2]. As a result, these women suffer from depression, irritability, and anxiety, with irritability being most frequently associated with functional impairment [3, 4]. In addition, it is estimated that among the 8–13% of women with a PMDD diagnosis, approximately 75% had a lesser diagnosis of PMS [5]. It is estimated that affected women suffer from almost 3000 days of discomforting symptoms throughout their reproductive years, with higher prevalence in some regions worldwide [6]. As a result, PMDD is an illness that carries a significant burden on society [7].

Currently, PMDD is pathophysiology rooted in impaired gamma-Aminobutyric acid type A receptors (GABAA-Rs) response to dynamic allopregnanolone (ALLO) fluctuations throughout the menstrual cycle [8, 9] in several brain areas such as the hippocampus amygdala, and prefrontal cortex [10]. Other key biomarkers of the condition include 5-hydroxytryptamine (5-HT), norepinephrine (NE), gamma-aminobutyric acid (GABA), progesterone (P), and Allopregnanolone (Allo) [11–13], but the exact mechanism is unknown. To explore the biological mechanisms of PMDD, reliable animal models are required. Previously, cycling rat PMDD models were studied for behavior and hormonal manipulations [10]. However, whether rats display aggression/irritability as a core symptom of PMDD remains an important question to be solved.

Robust studies have also indicated that selective serotonin reuptake inhibitors and oral contraceptives are taken early, as first-line drugs in treating PMS/PMDD are, to some extent, effective [14]. Alternative and complementary medicine have also been suggested as alternatives, including Vitex agnus castus (also called vitex, VAC) [15]. Apart from VAC, Baixiangdan in the form of capsules is an effective herb remedy for PMS/PMDD in our previous research [16]. To further elucidate the pharmacological mechanisms of Baixiangdan, we aimed to establish a PMDD rat model and evaluate its reliability as an animal model for PMS/PMDD-related research.

## MATERIALS AND METHODS

### Animals

Female Wistar rats, weighing 135 ± 15 g, were procured from the Beijing Weitong Lihua Experimental Animal Technology Co., Ltd, Beijing, China (License No. SCXK 2012–0001). After acclimatizing for a week at 21 × 1°C and 55% relative humidity, the animals were exposed to a 12 h/12 h light/dark cycle with reversed day and night (lights on at 20:00; lights off at 8:00). Water and food were available at all times.

### Estrous cycle screening

At the end of the acclimatization period, to detect the electrical impedance of the vagina, the RAT vaginal epidermal cell resistance test (EIV) was performed using a rat conception phase analyzer. A resistance value greater than 3Kohm from this test indicated that the rat was in the Proestrus stage. The test was conducted for a minimum of 15 days for each animal. Rats with regular motility cycles were selected for subsequent experiments.

### Resident-intruder paradigm (RIP)

After estrous cycle screening, rats with estrous cycle regularity of 4 or 5 days were selected as resident rats for RIP. The rats with smaller body sizes (−50 g as appropriate) were selected as Intruders. Bilateral ovariectomy was performed before the RIP test.

RIP was conducted in the breeding environment of resident rats from 14:30 to 17:30. First, intruder rats were put into cages of resident rats equipped with a video recorder set to record for 10 min. After 10 min, intruder rats were placed back into the new cages. All resident rats underwent RIP for 4 or 5 days (a complete physiological cycle). To avoid differences in individual fighting intensity, resident rats met different invading rats daily, designed by Latin squares, to ensure normal aggressive behavior. Meanwhile, the resistance of rat vaginal epidermal cells was tested daily in resident rats, and rats with irregular estrous cycles were removed at any time.

### Grouping

Mixed aggression scores of resident rats were calculated using a formula, and data from the RIP video was analyzed using an aggression analysis tool. Using the GraphPad Prism 9.4 statistical mapping software to analyze the scores, in the top 30% of the orders, rats were randomly assigned to the model group (model + fluoxetine, model + Baixiangdan), and rats in the bottom 30% were assigned to the control group.

### Behavioral phenotypes

#### Aggressive behavior test

An aggressive behavior test was performed according to the RIP measuring the irritability-like behavior [16].

#### Elevated plus maze (EPM)

The EPM was performed to measure anxiety-like behavior [17]. The super maze 3.0 animal behavior analysis system was used to record and track the free activity behavior of the rats within 5 min, including the entry time and times: to enter two open arms (OT), to enter two-arm seals (CT), to enter two open arms (OE) and to enter two closed arms (CE) (82), and in percentage to open arms OT% and in percentage OE%, as calculated using recorded results. The time was calculated using this formula: OT% = OT/ (OT + CT) × 100%, OE% = OE/(OE + CE) × 100%.

#### Open field test (OFT)

The OFT was performed to measure locomotor activity and anxiety-like behavior [18]. The rats were allowed to explore freely for 5 min in an open field box with a black bottom (length and width of 50 cm × 50 cm, and a 40 cm high wall). The open-field box and its surrounding areas were divided into nine palaces. The central area accounts for 1/9 of the entire open field, while the remaining are peripheral areas. The super maize 3.0 animal behavior analysis system was used to record and track the free activity behavior of rats within 6 min.

#### Breast width measurement

To perform breast width measurement [19], rats were anesthetized by intraperitoneal injection of chloral hydrate (10%) at 3 mL/kg. After completely unconscious, their limbs were spread out with binding lines, and their heads and bodies were tied to the rat plate. Appropriate anesthesia was determined by ensuring stable breathing, slow corneal reflex, relaxation of muscles in the whole body, and disappearance of skin reaction caused by hemostatic forceps. After which, depilatory cream was applied to the rat nipples using a cotton ball and allowed to sit for 2 min. The involved area of 1 cm^2^ was gently wiped off exfoliated hair with gauze soaked in normal saline to expose the skin and nipples. With a magnifying glass held in one hand and an electronic digital caliper in another, breast width measurement was taken close to the nipple.

### Hormone priming regimen

Ovariectomy and hormone priming were used to test whether the PMDD-like behavior phenotype was estrous cycle-dependent in the rats. Under anesthesia using pentobarbital sodium (60 mg/kg) 2%, the ovariectomy was performed after the behavioral phenotype test. Following a seven-day recovery period, an aggressive behavior test, an EPM, and an OFT were conducted during the D1 and P/E phases of the next estrus cycle. Next, the hormone priming regimen was performed according to a previous study [20]. During the second cycle of hormone priming, the above tests were performed.

### Drug treatment

Drug treatment was divided into two stages. As part of the first stage, rats were divided into control and model groups without being given any drugs. In the second stage, rats were divided into model, normal control, model + fluoxetine (2.7 mg kg^−1^ d^−1^) and model + Baixiangdan Capsules (0.2 g kg^−1^ d^−1^) groups.

### ELISA

After collecting behavioral indicators, rats in each group were anesthetized with chloral hydrate in the NR phase, and 3–5 mL of blood from the abdominal vein was collected. After being left standing for half an hour, the blood samples were centrifuged (3500 r/min, 15 min), and the supernatant was placed in tubes. The head of the rats was rapidly severed to collect brain tissue, which was immediately stripped off while on ice to avoid melting. The prefrontal cortex, hippocampus, and amygdaloid nucleus of the brain tissue samples were separated with curved tweezers. The samples were weighed and sub-packed into 1.5 mL tubes. The upper serum and three brain tissue samples were placed in a −80°C ultra-low refrigerator for subsequent examination. Next, samples from the plasma and brain tissues were used for ELISA analysis of GABA, 5-HT, NE, P, and ALLO using ELISA kits (GABA, 5-HT, NE, P, and ALLO) according to the instructions.

### Western blot analysis

Brain tissues were centrifuged (13000 rpm, 4°C, 10 min) in a lysate (PMSF: RIPA = 1:100) for tissue grinding. The resulting supernatant was collected and mixed with 5 × loading buffer into a 100°C water bath for 10 min for denaturation and stored at −20°C. Protein levels were determined using a BCA Kit (Solarbio, China; PC0020) according to the manufacturer’s instructions and spectrophotometry at 5.595 nm. After boiling for 5 minutes, samples (15 μL) were loaded onto 4% sodium dodecyl sulfate-polyacrylamide gel and separated by electrophoresis. After separation, the proteins were electrophoretically transferred to a polyvinylidene difluoride membrane and blocked in 5% non-fat milk at 25°C for 1 hour. After incubating the membrane with primary antibodies (rabbit polyclonal anti-GABRA4 antibody; Abcam, China; ab4120, and mouse polyclonal anti-β-actin antibody; Wuhan Sanying Biotechnology Co., Ltd, China; 66009-1-LG), secondary antibodies (HRP labeled Goat anti-mouse; Wuhan Sanying Biotechnology Co., Ltd, China; SA00001-1/2) were added. With the help of Image J software, the optical density of the positive protein bands was calculated using an enhanced chemiluminescence reagent (Solarbio, China; PE0010).

### RT-qPCR analysis

The total RNA was extracted from brain tissue using 1 mL Trizol reagent (Icosai Biotechnology, MB000) and reverse-transcribed into cDNA using ReverTra Ace qPCR RT Master Mix (Thermo Fisher Scientific, K1612). In order to perform RT-qPCR, dNTP mix (Solarbio, PC2200) was used, along with the Roche LightCycler 480 Real-Time PCR System. Data were analyzed using the Light Cycle 96 SW1.1 software by the 2^−ΔΔCt^ method. The primers used for RT-qPCR were synthesized by Sangon Biotech (China), and the sequences were as follows: GABAAR4α-F (5′–3′): GAAACCACTCCTAAGGCCCACT, GABAAR4α-R (5′–3′): GCGATGCGGCAGACGAAA, GAPDH-F (5′–3′): TCTCTGCTCCTCCCTGTTCT, GAPDH-R (5′–3′): TACGGCCAAATCCGTTCAC.

### Statistical analysis

Analyses were conducted using GraphPad Prism 9.4 and the mean ± standard error of the data was used to determine the distribution’s normality. Data from neurochemical and biochemical tests were analyzed using one-way ANOVA, followed by multiple comparisons using Tukey’s posthoc test. The level of significance was set at *p* < 0.05.

## RESULTS

### Behavioral characteristics of PMDD rat model induced by RIP

**Figure 1:**
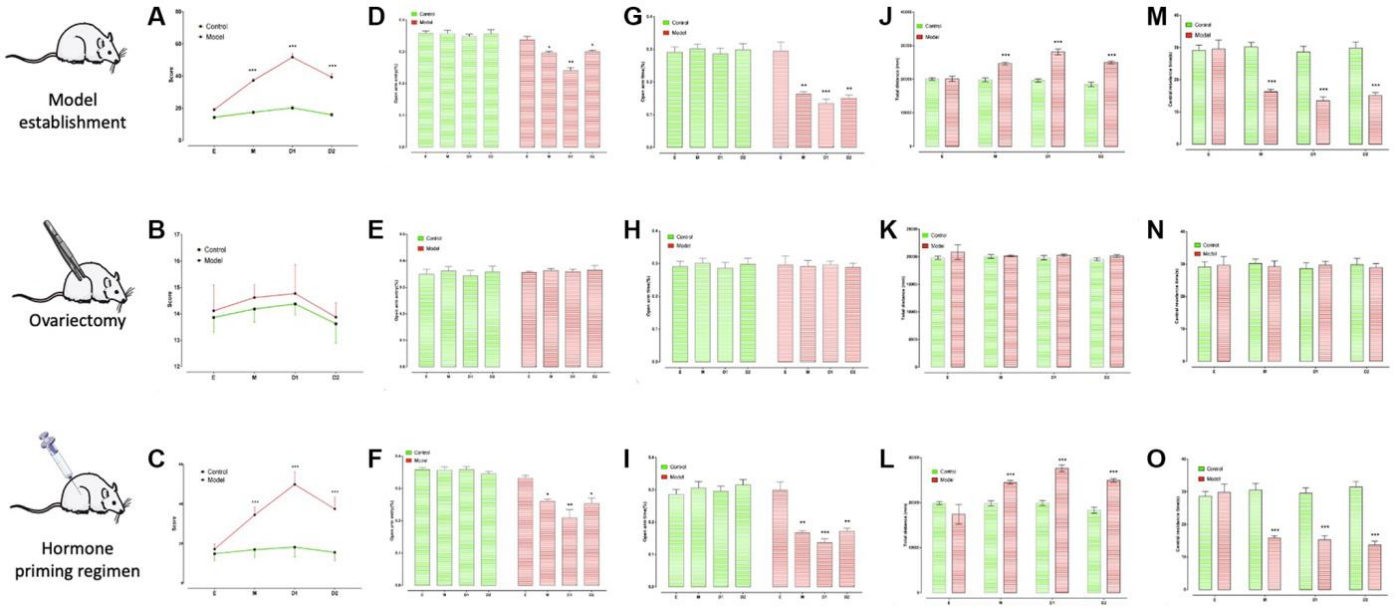
**Behavioral characteristics of PMDD model rats and their estrous cycle dependence identification.** (**E**) M, D1, and D2 represent the four stages of rats’ estrus. (**A**, **D**, **G**, **J**, and **M**) are behavioral characteristics of PMDD model rats under the state of the estrous cycle. (**B**, **E**, **H**, **K**, and **N**) are the behavioral characteristics of PMDD model rats losing estrous cycle after ovariectomy. (**C**, **F**, **I**, **L**, and **O**) are the behavioral characteristics of PMDD model rats after artificially induced estrous cycle recovery. (**A**) Reveals the score of mixed attacks before ovariectomy. (**B**) Shows the score of mixed attacks after ovariectomy. (**C**) Displays the score of mixed attacks after hormone induction. (**D**) Displays the percentage of the number of times to enter the open arm before ovariectomy; (**E**) Shows the percentage of the number of times entering the open arm after ovariectomy. (**F**) Reveals the percentage of the number of times entering the open arm after hormone induction. (**G**) Reveals the percentage of stay time in the open arm before ovariectomy. (**H**) Displays the percentage of stay time in the open arm after ovariectomy. (**I**) Displays the percentage of retention time in the open arm after hormone induction. (**J**) Represents the total distance before ovariectomy. (**K**) Represents the total distance after ovariectomy. (**L**) Represents the total distance after hormone induction. (**M**) Represents the residence time in the central area before ovariectomy. (**N**) Represents the residence time in the central area after ovariectomy. (**O**) Represents the residence time of the central area after hormone induction, ^*^p < 0.05, ^**^p < 0.01, ^***^p < 0.001.

Premenstrual irritability caused by RIP revealed the following behavioral phenotypes: PMDD rat models demonstrated slight aggressive behavior in the E phase and significant aggressive behavior in the M, D1, and D2 stages (especially D1) (Figure 1A). Simultaneously, the OT% and OE% of EPM in M, D1, and D2 phases (especially D1) decreased significantly (Figure 1D and 1G), and the total distance of OFT increased significantly (Figure 1J), and residence time in the central area decreased significantly (Figure 1M). These changes are a representation of apparent anxiety-like behavior.

### Estrous cycle-dependent verification of behavioral characteristics

Ovariectomy and hormone priming were used to test whether PMDD-like behavior phenotype was estrous cycle-dependent. Aggression test, EPM, and OFT analysis after ovariectomy revealed the disappearance of PMDD-like behavior phenotype (Figure 1B, 1E, 1H, 1K and 1N). However, after the hormone priming program was reestablished, which initiated the rat’s estrous cycle, PMDD-like emotional phenotype reappeared. The hormone program produced a significantly higher score of aggressive behavior in NR period than that in the normal control group. OE% and OT% were significantly lower in the hormone-primed group. In the normal control group, the distance to the central area of OFT was significantly increased, and residence time in the central area was significantly reduced (Figure 1C, 1F, 1I, 1L and 1O).

### Behavioral changes after PMDD drugs intervention

**Figure 2:**
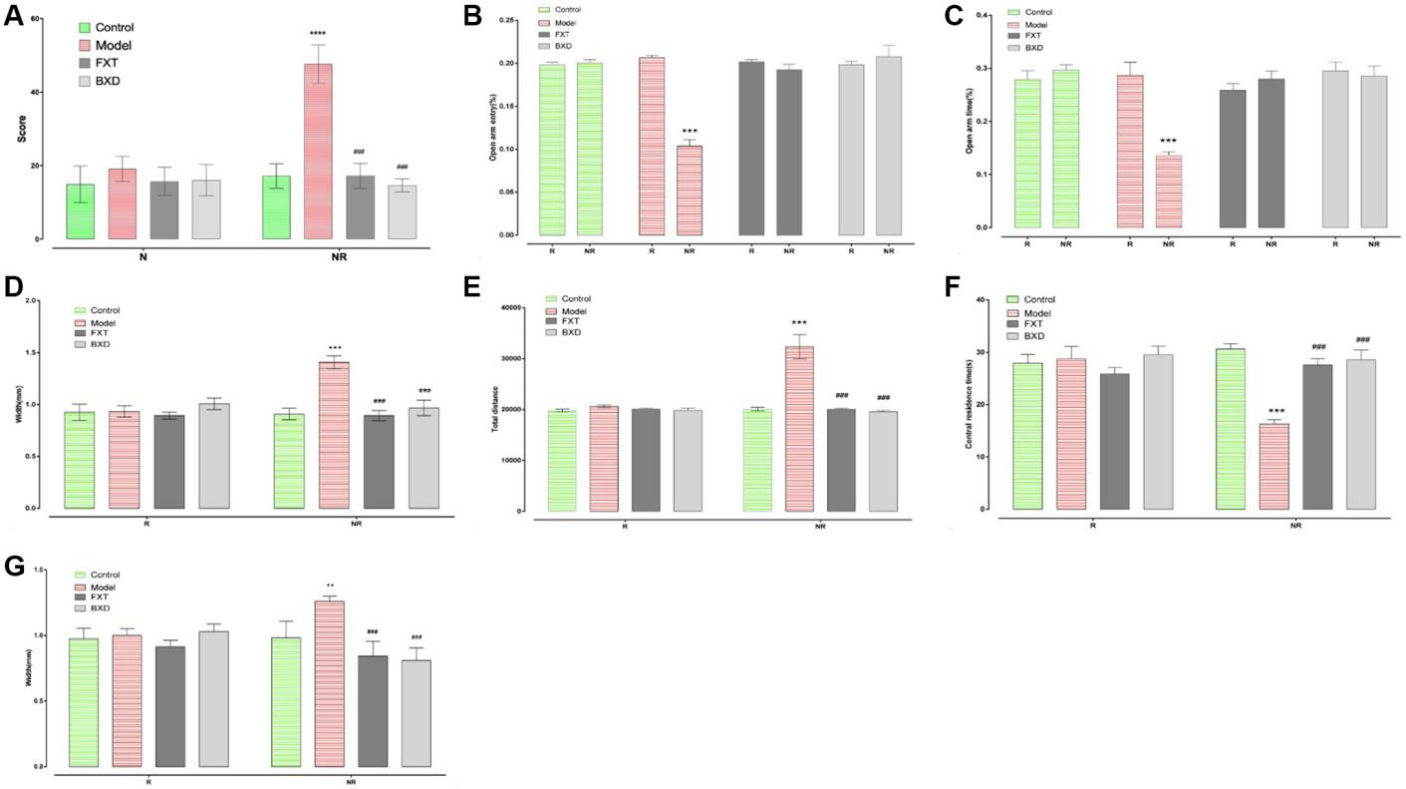
**Changes of model rats after drug intervention.** R stands for acceptance period experiment; NR stands for non-acceptance period experiment; (**A**) Shows a comparison of aggressive behavior test results after drug intervention; (**B**) Demonstrates the percentage of number of times that OE% of EPM enters the open arm after drug intervention; (**C**) shows OT% of EPM after drug intervention; (**D**) represents the total distance after drug intervention; (**E**) represents the central area residence time after drug intervention; (**F**) displays the left nipple width of rats; (**G**) displays the right nipple width of rats; ^*^p < 0.05 vs. control, ^**^p < 0.01 vs. control, ^***,#^*p* < 0.05 vs. model; ^##^*p* < 0.01 vs. model, ^##^*p* < 0.001 vs. model.

After fluoxetine and Baixiangdan intervened with two estrous cycles, the aggressive behavior disappeared and returned to normal levels (Figure 2A). In the treatment group, OE% and OT% increased (*p* < 0.001), and the total distance of OFT decreased (*p* < 0.001), and residence time in the central area increased (*p* < 0.001), which indicated that these drugs could reduce anxiety-like behavior in rats of this model (Figure 2B, 2C, 2E and 2F). The width between the left and right nipples was also found to be decreased (*p* < 0.001), which indicated alleviation of breast swelling (Figure 2D and 2G).

### Characteristics of hormone changes

**Figure 3:**
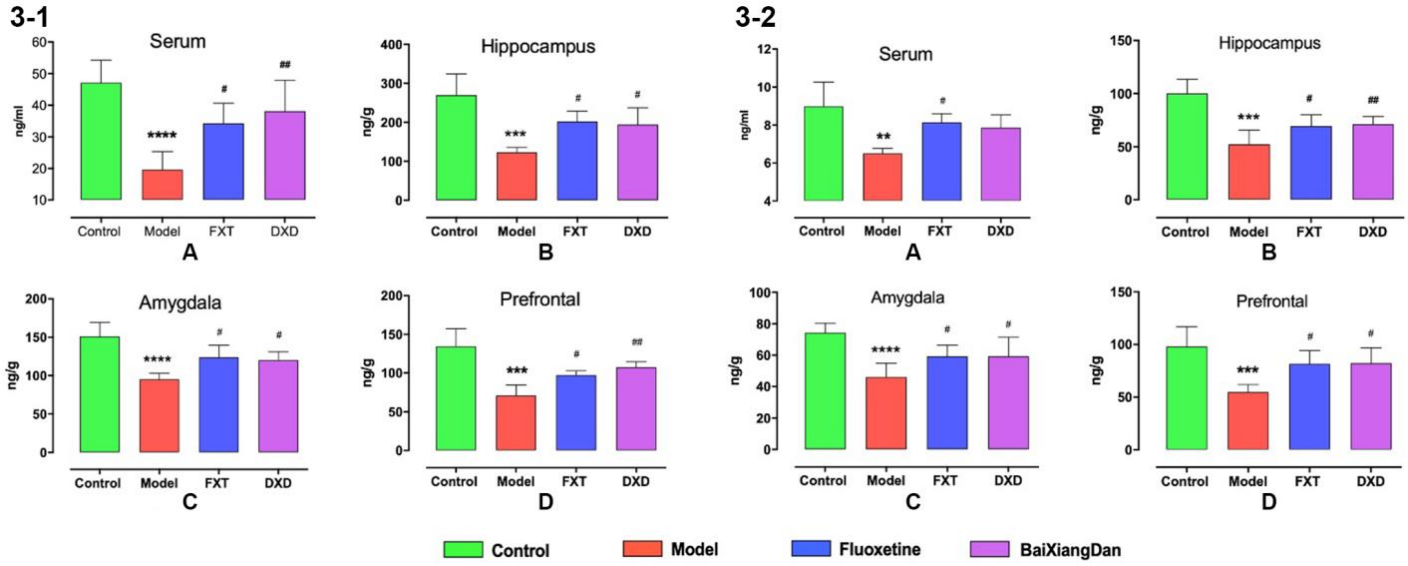
**Changes in P and ALLO contents in serum and brain of rats at various stages of the estrous cycle.** R represents the acceptance period, NR represents the non-acceptance period, (**3-1**) demonstrates changes in P content, and (**3-2**) shows changes to ALLO content, ^*^p < 0.05 vs. control, ^**^p < 0.01 vs. control, ^***,#^*p* < 0.05 vs. model; ^##^*p* < 0.01 vs. model, ^##^*p* < 0.001 vs. model.

ELISA determined the contents of P and ALLO in serum and brain. Compared with the normal control group, a significant decrease in P and ALLO contents in serum and the prefrontal lobe, amygdala, and hippocampus were noted in the model group (*p* < 0.01). Fluoxetine and Baixiangdan administration significantly increased the P content in serum and selected brain areas (*p* < 0.01) (Figure 3-1 and 3-2).

### Changes in neurotransmitter contents in PMDD premenstrual irritable rat model

**Figure 4:**
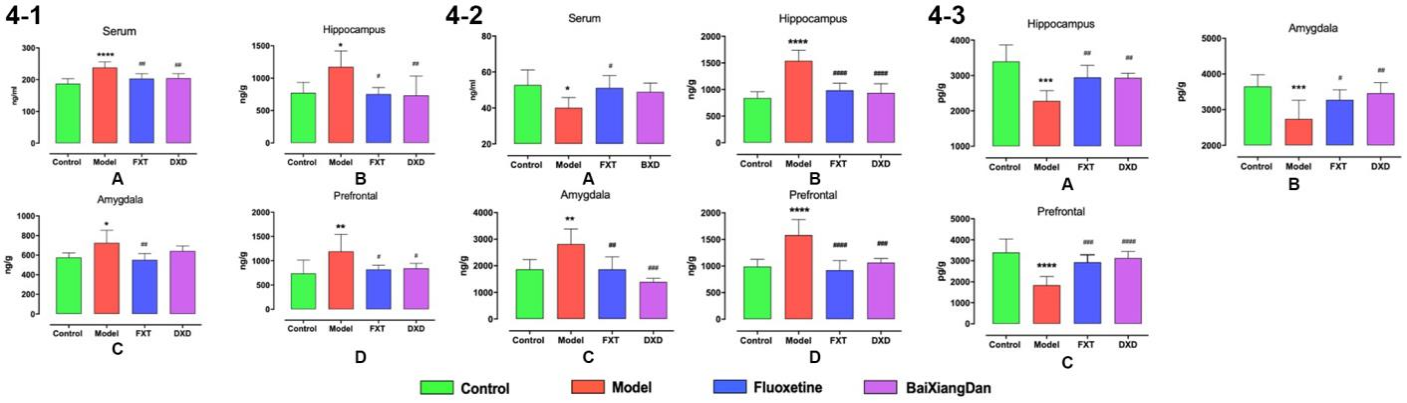
**Changes in neurotransmitters in serum and brain of rats at various stages of the estrous cycle.** R represents the acceptance period; NR represents the non-acceptance period, (**4-1**) displays the change of 5-TH content, (**4-2**) shows the change of NE content, and (**4-3**) demonstrates the change of GABA content, ^*^p < 0.05 vs. control, ^**^p < 0.01 vs. control, ^***,#^*p* < 0.05 vs. model; ^##^*p* < 0.01 vs. model, ^##^*p* < 0.001 vs. model.

ELISA also determined the contents of 5-HT, NE, and GABA in serum and brain. The level of 5-HT in serum, hippocampus, amygdala, and frontal lobe of model rats was significantly higher than in normal control rats (*p* < 0.05), and fluoxetine and Baixiangdan reduced this change to varying degrees (*p* < 0.05) (Figure 4-1). The content of NE in the brain of model rats was significantly higher than that of the normal control group (*p* < 0.05). Fluoxetine and Baixiangdan reduced this change to varying degrees (*p* < 0.05), but changes in NE in serum were contrary (Figure 4-2). Meanwhile, the GABA content in the hippocampus, amygdala, and frontal lobe of model rats was significantly lower than that of the normal control group (*p* < 0.005). Fluoxetine and Baixiangdan upregulated their levels (*p* < 0.05) (Figure 4-3). The regulating effect of Baixiangdan was close to that of fluoxetine.

### Gabaar4 in the brain of PMDD premenstrual irritable rats with α-type expression changes

**Figure 5:**
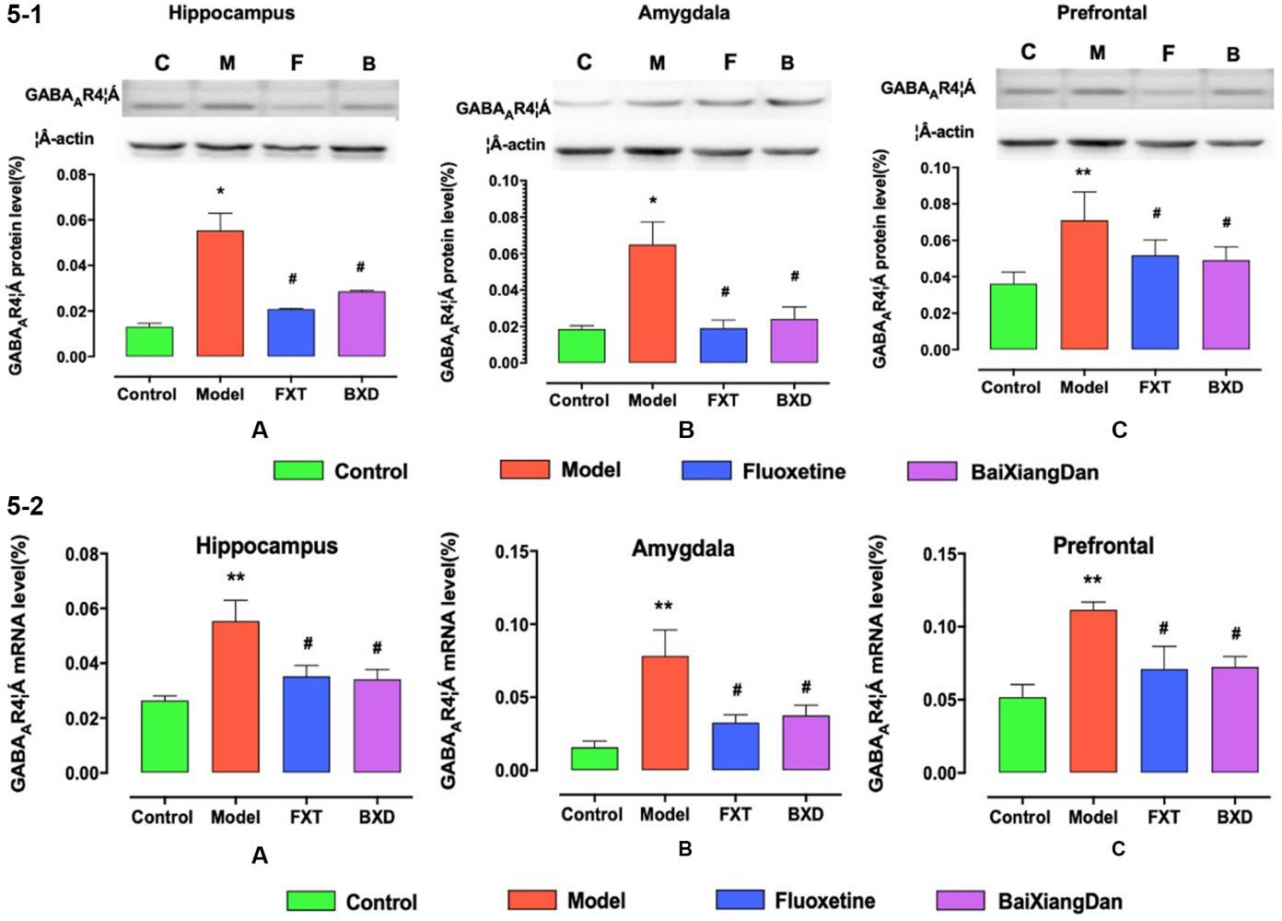
**GABAA4 expression levels in the hippocampus, amygdala, and frontal lobe.** (**5-1**) GABAA4 protein expression level in hippocampus, amygdala, and frontal lobe. (**5-2**) GABAA4 mRNA expression level in hippocampus, amygdala, and frontal lobe. ^*^p < 0.05 vs. control, ^**^p < 0.01 vs. control; ^#^*p* < 0.05 vs. model.

The expression levels of GABRA4 mRNA and GABRA4 protein in the brain were quantitated using real-time polymerase chain reaction (RT qPCR) and Western blot, respectively. Figure 5-1 displays a similar increasing trend of GABRA4 in model rats’ hippocampus, amygdala, and frontal lobe. The protein expression in the model group was significantly higher than in the control group (*p* < 0.01), and the expression was significantly reduced after the administration of drugs (*p* < 0.05). Figure 5-2 shows the same protein expression changes of GABAAR4α at the mRNA and protein levels in the hippocampus, amygdala, and frontal lobe. Similar to the protein, the mRNA expression level was significantly higher in the model group (*p* < 0.05) with a significant reduction after drug treatment (*p* < 0.05).

## DISCUSSION

Recently, studies focusing on the neurobiology of PMDD evidenced the complexity of its diagnostic and management [21–23]. Animal rat models have been previously created to study some core symptoms of PMDD, including depressive-, irritability/aggressive- and anxiety-like behaviors [20, 24]. However, recently, the resemblance of the animal models to premenstrual dysphoric conditions has been queried [10]. In this study, we used methods mimicking premenstrual dysphoric conditions in rats. Specifically, RIP was used to induce rats to show PMDD-like behavior, ovariectomy to test the relationship between symptoms and estrous cycle, fluoxetine and Baixiangdan treatments to test the reversal effect of PMDD-like behavior, biomarkers to detect internal pathological mechanisms, and clinical connectivity measurement to verify the degree of simulation of PMDD in a PMDD rat model.

A previous study [22] referred to optimizing RIP as a modeling method. Compared with normal rats, RIP-induced rats demonstrated slight aggressive behavior in phase E, and significant aggressive behavior was depicted in phase D1, successfully simulating irritability in clinical patients. At the same time, the OT% and OE% of EPM in the D1 phase were significantly reduced, the total distance of OFT was significantly increased, and residence time in the central area was significantly reduced, all of which simulate anxiety-like emotions commonly experienced by clinical patients (Figure 1).

At present, OFT, EPM, and RIP are standard tests for evaluating the behavior of animal models of PMDD [25–27], while RIP is still a standardized method to measure aggression and anger in a semi-natural setting [28]. The behavior demonstrated by rats in the current PMDD model, including the aggression test, was consistent with published findings [16, 26]. Furthermore, a core feature of PMDD is the periodically changing symptoms based on the menstrual cycle. Thus, ovarian suppression or ovariectomy was often effective for some patients with PMS [29–31]. This study performed a series of related trials involving ovariectomy and hormone priming to verify our models’ reliability. The disappearance of PMDD-like behavior after ovariectomy and the appearance of PMDD-like behavior after the hormone priming regimen confirmed the relevance of our model to the estrous cycle, successfully reflecting the pathogenesis of PMDD [10, 32]. Another test involved the usage of drugs.

Fluoxetine is FDA-approved for premenstrual dysphoric disorder and recommended by doctors in clinics [33, 34], and frequently used as a positive drug in animal experiments [35]. Moreover, as an alternative to pharmacological therapy, herbal medications are often used for gynecological disorders [36]. Baixiangdan in capsules is an anxiolytic Chinese patented medicine for the treatment of PMDD with proven efficacy [16, 37]. After administration of both medications, PMDD rat models was reversed and improved (Figure 2), which are similar outcomes seen with PMDD patients under the treatments. Furthermore, biomarker characteristics and their pharmacological changes were studied, particularly P, ALLO, and GABA-related molecules, which are associated with GABAARs’ response to dynamic ALLO fluctuations. Figure 3 display that rats demonstrated PMDD-like behavior with decreased P and ALLO contents. Concurrently, 5-HT, NE, and GABA contents also had specific changes, while the expression level of GABRA4 specifically increased. Fluoxetine and Baixiangdan reversed these changes in the model rats. The above outcomes verified the model’s validity and indicated the drugs’ mechanism of action, consistent with other relevant research findings [38–40].

Nevertheless, some biological indicators such as liver-qi invasion and liver-qi depression syndromes of PMDD based on TCM theories proposed by Qiao et al. [41] were inconsistent with another PMDD model [20]. The inconsistencies suggest the possibility of the existence of PMDD subtypes [42]. Another observation was found in our previous study that may contribute to the differences, whereby PMDD patients were prone to anger and depression but had different neural mechanisms in emotional processing than those without PMDD [43].

## CONCLUSION

RIP successfully induced premenstrual irritability in rats of this PMDD model, and its behavioral phenotype was consistent with the clinical symptoms of PMDD. Fluoxetine and Baixiangdan demonstrated excellent therapeutic effects on premenstrual irritability, possibly by increasing the content of P and GABA in serum and regulating the abnormal fluctuation of 5-HT and E^2^ levels. Meanwhile, the levels of 5-HT, NE, and protein and mRNA expression of GABRA4 increased. The reduction of GABA, P and ALLO may be the critical biomarkers of abnormal change in the pathogenesis of PMDD.

### Strengths and limitations

Based on the improvement of existing methods, this study proposes a novel rat model of PMDD and provides sufficient data support. This rat model can simulate the symptoms of PMDD to a large extent, which is of great significance for the research on the pathogenesis of PMDD and drug development. However, due to insufficient detection methods, the simulated animal models for some physical symptoms of PMDD cannot be accurately detected, and further in-depth research should be carried out in the future.
